# Exploring the transcription activator-like effectors scaffold versatility to expand the toolbox of designer nucleases

**DOI:** 10.1186/1471-2199-15-13

**Published:** 2014-07-05

**Authors:** Alexandre Juillerat, Marine Beurdeley, Julien Valton, Séverine Thomas, Gwendoline Dubois, Mikhail Zaslavskiy, Jérome Mikolajczak, Fabian Bietz, George H Silva, Aymeric Duclert, Fayza Daboussi, Philippe Duchateau

**Affiliations:** 1 CELLECTIS S.A, 8 Rue de la Croix Jarry, Paris 75013, France

**Keywords:** Transcription activator-like effectors, TALE, TALEN, Protein engineering, Genome editing

## Abstract

**Background:**

The past decade has seen the emergence of several molecular tools that render possible modification of cellular functions through accurate and easy addition, removal, or exchange of genomic DNA sequences. Among these technologies, transcription activator-like effectors (TALE) has turned out to be one of the most versatile and incredibly robust platform for generating targeted molecular tools as demonstrated by fusion to various domains such as transcription activator, repressor and nucleases.

**Results:**

In this study, we generated a novel nuclease architecture based on the transcription activator-like effector scaffold. In contrast to the existing Tail to Tail (TtT) and head to Head (HtH) nuclease architectures based on the symmetrical association of two TALE DNA binding domains fused to the C-terminal (TtT) or N-terminal (HtH) end of FokI, this novel architecture consists of the asymmetrical association of two different engineered TALE DNA binding domains fused to the N- and C-terminal ends of FokI (TALE::FokI and FokI::TALE scaffolds respectively). The characterization of this novel Tail to Head (TtH) architecture in yeast enabled us to demonstrate its nuclease activity and define its optimal target configuration. We further showed that this architecture was able to promote substantial level of targeted mutagenesis at three endogenous loci present in two different mammalian cell lines.

**Conclusion:**

Our results demonstrated that this novel functional TtH architecture which requires binding to only one DNA strand of a given endogenous locus has the potential to extend the targeting possibility of FokI-based TALE nucleases.

## Background

**Figure 1:**
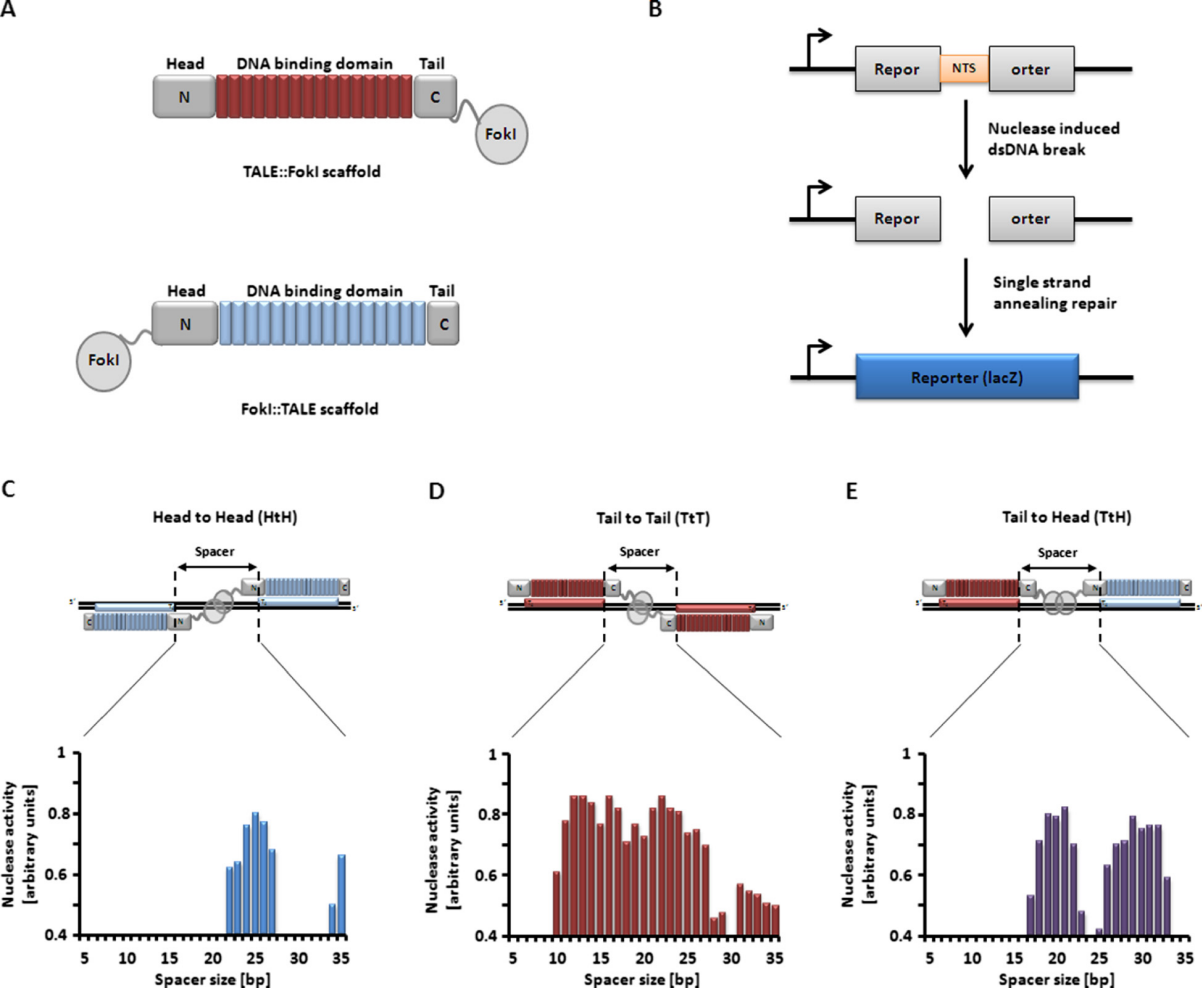
**Design, creation and *In vivo *characterization of the three nuclease architectures based on the FokI catalytic domain in yeast. (A)** Schematic representation of the two different scaffolds used in this study including the positions of N and C-terminal domains, DNA binding domain as well as the Tail and Head positions. **(B)** Schematic representation of the yeast extrachromosomal single strand annealing (SSA) assay. The reporter plasmid containing a Nuclease Target Sequence (NTS) is flanked by overlapping truncated LacZ genes sequences. Cleavage of the target sequence in yeast leads to the restoration of the LacZ marker through the single strand annealing (SSA) pathway of recombination. The restoration of the functional LacZ gene is quantified by a β-galactosidase activity assay and related to the nuclease efficiency. **(C, D and E)** Representative examples of activity measurements from the yeast SSA assay for the three architectures obtained on the same filter. **(C)** HtH architecture where two FokI::TALE scaffolds are facing each other on the two DNA strands and in a head to head orientation. **(D)** Classical TtT architecture where two TALE::FokI scaffolds are facing each other on the two DNA strands and in a Tail to Tail orientation. **(E)** TtH architecture where a TALE::FokI and a FokI::TALE scaffolds are facing each other on the same DNA strand and in a Tail to Head orientation. The nuclease activity measured for the three architectures in yeast using the single strand annealing assay (SSA) as a function of target spacer length (5-35 bp) is displayed at the bottom of each figure panels. For each filter, three controls (negative control, weak nuclease and strong nuclease) were measured multiple times (n > 100). Standard deviation on these activity measurements were typically of 0.05.

Transcription activator-like effectors (TALEs), a group of bacterial plant pathogen proteins, have recently emerged as new engineerable scaffold for production of engineered DNA binding domains with chosen specificities [reviewed in [1]]. The targeting specificity of this family of proteins is driven by a central core composed of multiple repeated units. These 33 to 35 amino acids repeated units are nearly identical to one another except for two polymorphic amino acids called RVDs (repeat variable di residue), responsible for the specific recognition of a unique nucleotide [2,3]. In addition to this central core domain, the N-terminal domain of TALE has been reported to play a key role in TALEs specificity and binding mechanism. This domain displays a strong specificity bias toward a thymine nucleotide, the so called “T0”, systematically located at the 5’end of the TALE target [2]. These different biochemical features were confirmed by the high resolution structure of TALE/DNA complexes, illustrating how the TALE protein wraps around its DNA target, from the 5’ T0 to the 3’ last nucleotide, in an N- to C-terminal orientation [4-6]. The particular DNA binding properties of TALE DNA binding domain, their exquisite specificity as well as their modularity have been used to develop engineered TALE nucleases named TALEN with tailored DNA specificity. The original TALEN architecture developed by Christian *et al*. [7], consisted of a custom TALE DNA binding domain linked to the N-terminal end of the non-specific FokI nuclease domain (TALE::FokI scaffold, Figure 1A). Because FokI needs to dimerize to catalyze a double strand break (DSB), TALEN work by pairs. Each pair unit binds in a Tail to Tail (TtT) orientation to adjacent binding sites, starting by a 5’ T0 and respectively located on the sense and antisense strand of the DNA. Such symmetrical architecture, originally designed to respect (i), the natural organization of the endonuclease and DNA binding domains of FokI (ii), the orientation of TALE DNA binding and (iii), the requirement of a T0, led to an incredibly robust TALE-based nuclease platform. Considered as the gold standard TALEN architecture, it has been extensively optimized and used for different gene editing applications [8,9]. Furthermore, the versatility of the TALE scaffold was demonstrated by the accumulation of studies reporting fusion (N-terminal as well as C-terminal) of the TALE core to various catalytic domains [10-21]. In particular, we [22] and others [23] have reported the development of TALE-based nuclease with an N-terminal fusion FokI catalytic domain.

In this study, we further explored and exploited the versatility of the TALE scaffold to develop a novel asymmetrical hybrid TALE and FokI-based nuclease architectures, the Tail to Head (TtH) architecture referred herein as TtH. We demonstrated the potential of this architecture to generate targeted mutagenesis at different endogenous loci in mammalian cells. In contrast to the conventional TALEN architecture, the TtH architecture only required one DNA strand of a given locus to efficiently bind and process it. Thus, our work presents a new advance in the development of TALE nucleases and further extends their targeting possibilities.

## Results

### Design and evaluation of the Head to Head (HtH) symmetrical architecture in a yeast SSA assay

To investigate the versatility of the TALE scaffold and generate alternative nuclease architectures, we used a yeast-based nuclease activity assay [24]. This assay was previously demonstrated to be suitable to assess the intrinsic nuclease activity of TALE nuclease without being biased by epigenetic modifications or chromatin context. In addition, we have previously found a good correlation between data obtained with the yeast SSA assay, an extrachromosomal SSA assay in CHO-K1 and chromosomal disruption experiments in CHO-KI [25,26]. We thus believed that the yeast model system could serve as an appropriate and representative assay to compare characteristics of different nuclease architectures. This assay relies on two yeast strains, one expressing the nuclease of interest and the other the target sequence flanked by overlapping truncated LacZ genes (Figure 1B). After mating of the two strains, the restoration, upon target cleavage, of the LacZ marker though the SSA pathway of recombination recontitute a functional LacZ gene. The resulting β-galactosidase can further be quantified and related to the nuclease efficiency. The experimental conditions were optimized to avoid saturation of the signal, thus allowing a direct comparison on the whole range of activities.

In order to evaluate alternative configurations to the standard TtT TALEN (symmetrical association of two TALE::FokI scaffolds, Figure 1A), we designed a construction harboring a FokI catalytic domain fused to the N-terminal domain of a TALE (FokI::TALE scaffold), leading to the symmetrical head-to-head nuclease architecture referred herein as HtH (Figure 1C). Throughout this study we used the avirulence protein AvrBs3 as scaffold (accession number P14727). The first step for developing the alternative HtH and TtH architectures was to re-engineer a fusion protein to create an efficient FokI::TALE scaffold. Early works performed on TALE protein showed that the first 152 amino acids of the N-terminal domain could be deleted (Δ152 variant) without affecting the protein activity [27]. We used an approach previously described to create an active I-TevI based TALE nuclease [22] and fused the FokI catalytic domain (amino acids 388 to 583, accession number P14870) to the N-terminal end of the Δ152 TALE variant via a 4 aminoacids (-GSSG-) flexible linker. In addition, we removed most of the C-terminal end of the TALE domain (keeping only the first 11 amino acids, amino acids 887 to 897, accession number P14727) to minimize the global size of the final protein. To allow the specific targeting of the desired DNA sequences, we used the canonical RVD/nucleotide association code (NI:A, HD:C, NN:G and NG:T) [2,3].

A RVD array targeting an 18 base pairs sequence of interest (ATATAAACCTAACCCTCT, Additional file 1: Table S2) was cloned into the FokI::TALE backbone. The nuclease activity of the resulting construction was tested in yeast using the extrachromosomal single strand annealing (SSA) assay (Figure 1B) and homodimeric DNA targets containing respectively two identical recognition sequences juxtaposed with the 5’ ends proximal (Figure 1C). To determine the optimal distance for cleavage activity between the two recognition domains, a series of homodimeric targets were designed with spacers ranging from 5 to 35 bp (Figure 1C, Additional file 1: Table S2). Furthermore, we also prepared the classical TtT TALEN targeting the same DNA sequence to serve as a reference for the currently used architecture (Figure 1D). In this study, we used a + C40 (amino acids 887 to 926, accession number P14727, followed by a 4 aminoacids –ISRS- linker) TALEN scaffold as, in our hands, this truncation presented a good balance to obtain a high activity associated with a good specificity (narrow spacer window). We additionally designed series of homodimeric targets containing respectively two identical recognition sequences juxtaposed with the 3’ ends proximal (Figure 1D, Additional file 1: Table S3) with the same spacing described above. The results obtained from this SSA assay showed that, despite not preserving the natural N-terminus (DNA binding domain) to C-terminus (catalytic domain) layout of the wild-type FokI, similar levels of activity for both architectures can be obtained (Figure 1C and 1D). Interestingly, one major difference between the two configurations was the spacing pattern reached by the HtH architecture, with a much narrower window of cleaved spacers compared to the ones obtained for the classical architecture (22 to 27 bp for the HtH nuclease versus 10 to 27 bp for the TtT nuclease).

### Design and evaluation of the Tail to Head (TtH) asymmetrical architecture in a yeast SSA assay

Having demonstrated that the FokI::TALE scaffold display a high nuclease activity in a symmetrical HtH configuration, we next evaluated its ability to pair up with the TALE::FokI fusion scaffold and produce an active nuclease (Additional file 1: Figure S1E). The nuclease activity of the resulting asymmetric tail-to-head architecture, referred herein as TtH, was assessed in yeast on a collection of hybrid asymmetric targets. These targets contained two different recognition sequences juxtaposed with the 3′-5′ ends proximal and separated by a DNA spacer ranging from 5 to 35 bp (Figure 1E, Additional file 1: Table S4). The yeast SSA assay results showed activity levels comparable to the ones observed for TtT and HtH architectures, with two distinct windows of cleaved DNA spacers (18 to 22 bp and 27 to 33 bp; Figure 1E). This result suggested an optimal cleavage every one helix turn of DNA. Interestingly, the optimal cleavage distance of TtH architecture was 5 bp shorter than the one obtained for HtH architecture (20 and 25 bp respectively).

### Nuclease activities of TtH asymmetrical architecture in mammalian cells and molecular characterization of nuclease-induced events

Once the activity of the new TtH architecture was demonstrated in yeast, we next investigated its activity in mammalian cells. Two loci of interest for potential therapeutic applications, previously chosen to investigate other tailored-made nucleases [25], DMD (gene involved in the Duchenne Muscular Dystrophies) and RAG1 (V(D)J recombination-activating protein 1), were selected in the human genome and an additional locus of interest for bioproduction, the fucosyltransferase 8 (FUT8) gene, was chosen in the Chinese hamster genome.

**Figure 2:**
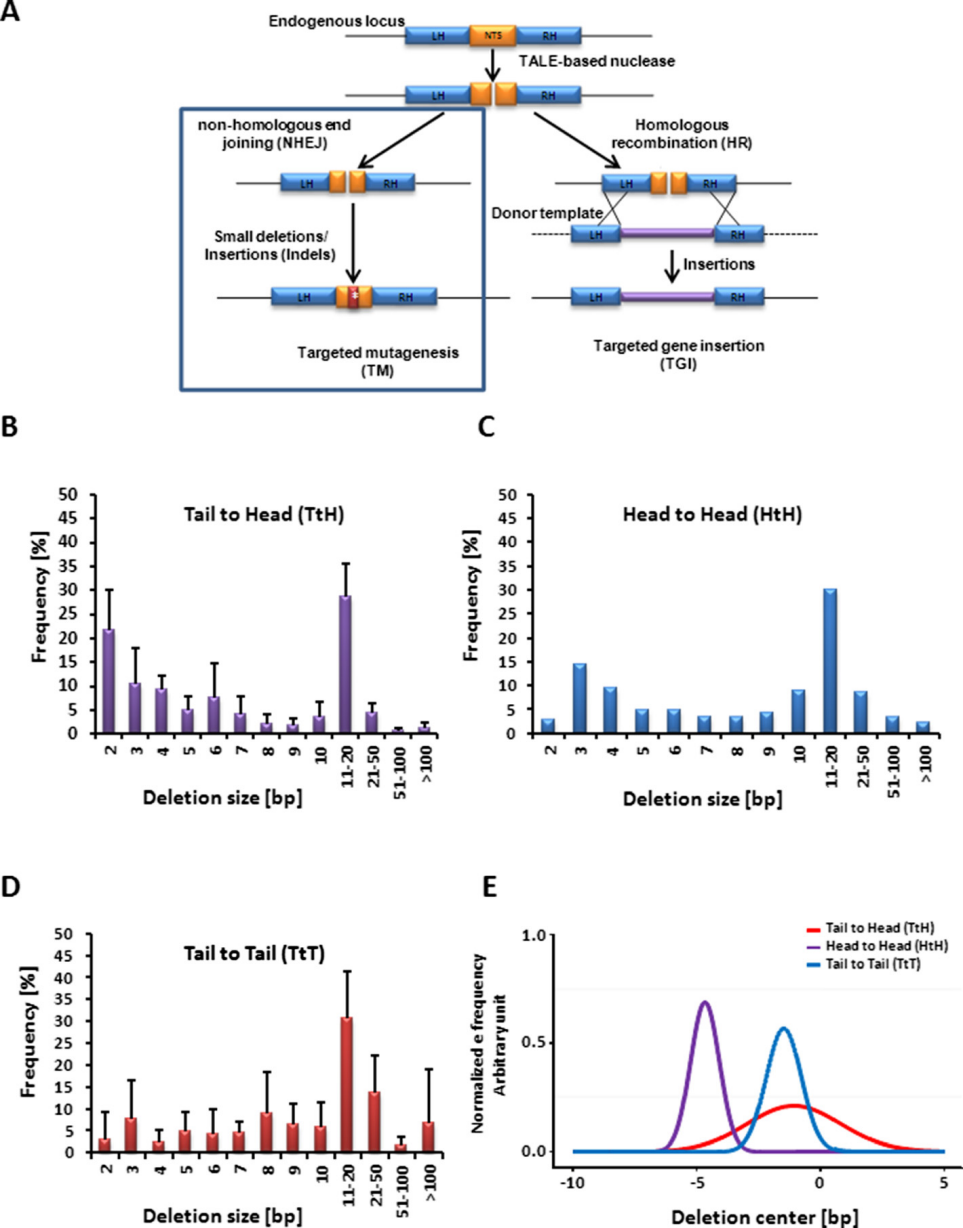
***In vivo *nuclease activity of the three nuclease architectures in mammalian cells. (A)** Schematic representation of nuclease-mediated gene inactivation via the error-prone NHEJ pathway. **(B)** Size distribution of the deletion events induced at the endogenous locus by the TtH architecture. Loci presenting at least 20 events were taken into account to generate the figure. Error bars denote s.d. Student t test performed to compare deletion patterns induced by TtT and TtH architectures showed no statistical difference (p-value = 0.3852). **(C)** Same as for **(A)** but for the HtH nuclease architecture. One locus presenting at least 20 events was used. **(D)** Same as for **(A)** but for the TtT nuclease architecture. **(E)** Representation of the localization of the deletion center for the three architectures. The Gaussian curves having the same mean and variance of deletion centers for each of the three TtH, HtH, and TtT architectures are represented. The areas under the curves have been normalized to 1. *t-test*, p-value = 0.00155 with respect to the TtT architecture. For the TtT and HtH architectures, due to the odd number of nucleotides present in their spacer (15 or 25 respectively), we arbitrary chose to place the center of the spacer at 8 or 13 bp, explaining the shift of the deletion center close to -1. Data from 3 loci (DMD, FUT8 and RAG) were used for the HtH and TtH architectures. Data from 10 loci (APC, MLH, CD52, NR3C3, LIG4, BBC3, NR3C2, M2K, PPARD, ERBB2) were used for the TtT architectures.

Because the nuclease activity of HtH architecture towards endogenous loci had never been reported, we also characterized this architecture in the following experiments. Targets were selected for both nuclease architectures according to the spacer profile (25 bp and 20 bp for HtH and TtH respectively) determined previously with the yeast SSA assay (Figure 1C and 1E). The nucleases were then assembled using the optimal scaffolds containing an additional N-terminal SV40 nuclear localization sequence to improve their *in vivo* nuclear targeting (Additional file 1: Table S5). These nucleases were then assayed for their ability to promote targeted insertions or deletions of nucleotides (indels) via error prone non-homologous end joining (NHEJ, Figure 2A), in the adequate cell-line (293H or CHO-KI, Table 1).

**Table 1:** Activities of the TtH and HtH nuclease architectures at their endogenous cognate targets

** Architectures and endogenous loci**			**Total events [%]**	**Total events [nb]**	**Insertion [nb]**	**Deletion [nb]**	**Wt [nb]**	**Reads [nb]**
**TtH**	DMD	NC_000023.10: 32,364,567-32,364,620	1.8	193	58	141	10538	10731
					(16)	(48)		
**ctrl**	DMD	NC_000023.10: 32,364,567-32,364,620	0	0	0	0	7837	7837
**TtH**	RAG1	NC_000011.9: 36,594,622-36,594,675	2.6	59	18	41	2221	2280
					(7)	(24)		
**ctrl**	RAG1	NC_000011.9: 36,594,622-36,594,675	0	0	0	0	5494	5494
**TtH**	FUT8	NW_003613860.1 673,480-673,533	8.7	459	134	330	4817	5276
					(60)	(80)		
**ctrl**	FUT8	NW_003613860.1 673,480-673,533	0.033	2	1	1	6129	6131
**HtH**	DMD	NC_000023.10: 32,364,534-32,364,592	0.01	1	1	0	9147	9148
**ctrl**	DMD	NC_000023.10: 32,364,534-32,364,592	0.01	1	1	0	9540	9541
**HtH**	RAG1	NC_000011.9: 36,594,571-36,594,629	1.2	10	5	6	861	871
					(3)	(6)		
**ctrl**	RAG1	NC_000011.9: 36,594,571-36,594,629	0.051	2	1	1	3913	3915
**HtH**	FUT8	NW_003613860.1: 673,442-673,500	30.5	1791	1191	613	4088	5879
					(64)	(159)		
**ctrl**	FUT8	NW_003613860.1: 673,442-673,500	0	0	0	0	7223	7223

The number of unique events (considering the size and position) is indicated in brackets. Control (ctrl) indicates a tranfection with an empty vector plasmid.

Three days post transfection, genomic DNAs were recovered and amplified by locus specific PCRs (370 to 630 bp). PCR amplicons were then analyzed by deep sequencing to determine the amount of Indels promoted by the different nucleases at their respective target site. Deep sequencing analysis demonstrated that two out of the three HtH nucleases displayed significant levels of targeted mutagenesis (2% and 35% of mutagenesis frequencies, Table 1). For the TtH conformation, all three nucleases showed activity on their respective target sequence, with Indel frequencies ranging from 2 to 9% (Table 1). However, the level of targeted mutagenesis generated by these two architectures (HtH and TtH displayed respectively 12% and 5% mean Indels frequencies) was lower than that reported for the classical TtT in two large scale studies (22% and 16% mean Indel frequencies) [28,29].

We next compared the NHEJ-dependent molecular events promoted by the nuclease activity of the two different architectures versus the classical TtT. Toward this goal, we generated 10 TtT TALEN, performed targeted mutagenesis experiments in 293H cells and recovered the resulting deep-sequencing dataset (Additional file 1: Table S6 and S7). We first compared the deletion length induced by the three different architectures and found similar patterns (p-value = 0.3852) with a large proportion of deletions smaller than 20 bp (Figure 2B-D), a feature previously described for the conventional TtT architecture [30]. However the important error bars obtained for some deletion sizes indicated a variability of DNA repair outcomes from one locus to another. Such variability could be due to several parameters including the RVD composition (DNA binding affinities) and the presence of micro-homologies in the targeted locus.

**Figure 3:**
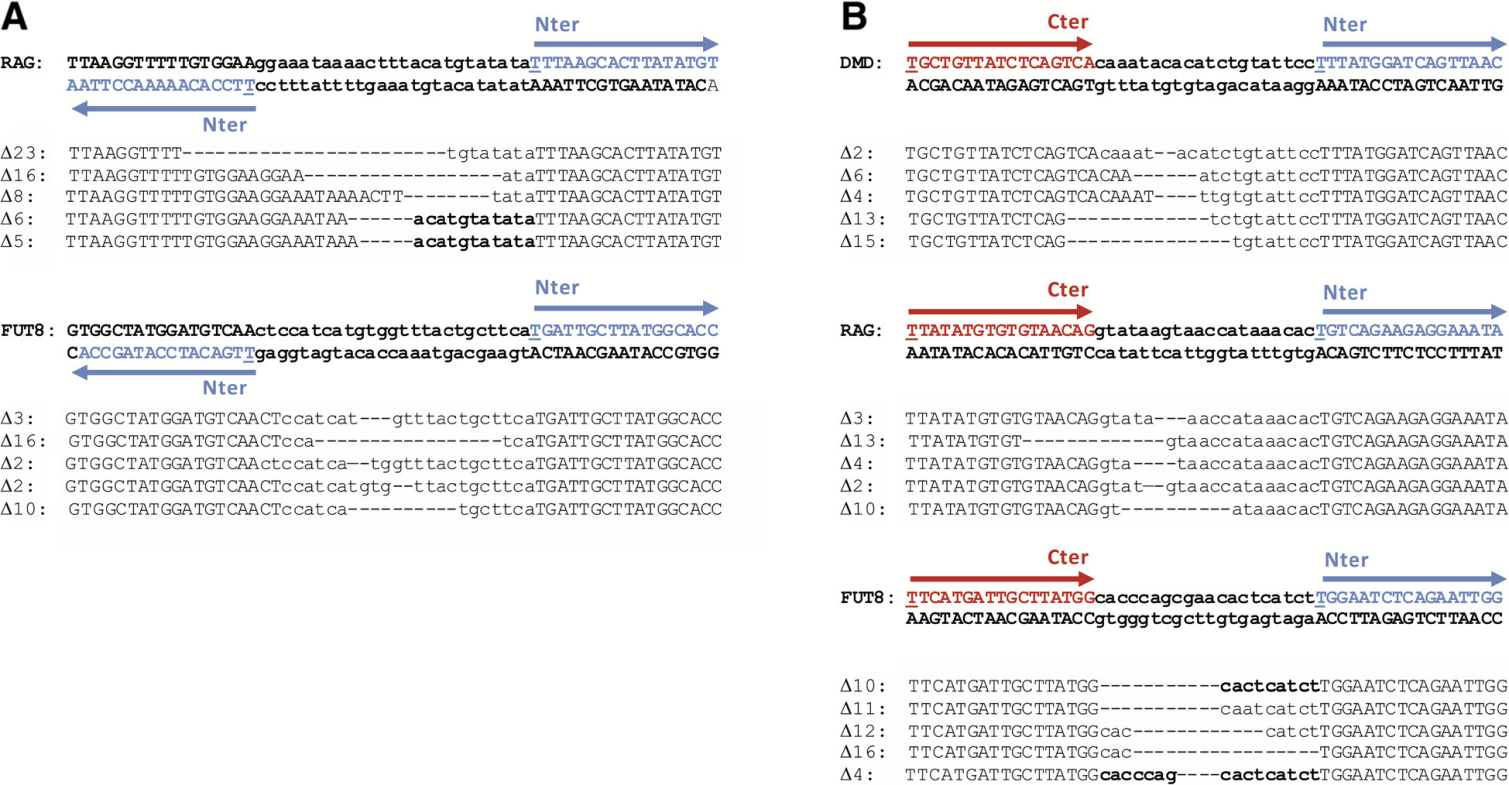
***Characterization of mutagenic events promoted by *the HtH and TtH nuclease architectures in mammalian cells. (A)** Alignment of the WT genomic sequence and predominant deletion events induced by the HtH architecture at two different endogenous loci. FokI::TALE scaffold binding sites are represented by capital letters. **(B)** Same as **(A)** but for the TtH architecture at the three different endogenous loci. The targeted sequence is colored with respect to the scaffold binding site considered (blue: FokI::TALE, red: TALE::FokI). The position 0 is underlined.

We then compared the position of mutagenic events within the spacer of each architecture target. Considering the fact that the optimal distance of cleavage was different for the two TtT and HtH symmetrical architectures, we hypothesized that the position of mutagenic events within the target spacer of TtH asymmetrical architecture would be eccentric. Interestingly, a statistical analysis of the deletion profiles revealed that its activity led to a significant shift of the deletion pattern (*t-test*, p-value = 0.00155 with respect to the TtT architecture) towards the TALE::FokI binding site (Figure 2E, Figure 3A and B). Due to their symmetrical configuration, the TtT and HtH architectures were expected to cleave right in the middle of their optimal target (Figure 1B) and thus, 7 to 8 bp and 12 to 13 bp away from the 3’ end of their respective FokI::TALE and TALE::FokI binding sites. However, due to its asymmetrical configuration, the TtH architecture was rather expected to cleave 2 to 3 bp away from the middle of its optimal target. The consistency between our experimental data and theoretical expectations indicated that the position of cleavage catalyzed by the TtH nuclease is constrained by its FokI::TALE scaffold component.

## Discussion

In this paper, we explored the versatility of the TALE scaffold and exploited it to generate a novel asymmetric FokI-based TALE nuclease architecture. This architecture called TtH TALE nuclease consisted of the asymmetrical association of TALE::FokI and FokI::TALE scaffolds (Figure 1A), two different engineered TALE DNA binding domains fused to the N- and C-terminal ends of FokI nuclease domain. Its nuclease activity was characterized in yeast toward extrachromosomal surrogate targets as well as in mammalian cells at different endogenous loci. Our results showed that this architecture was active in yeast and further allowed to promote targeted mutagenesis at multiple endogenous loci in mammalian cells. Importantly, the TtH architecture only required one DNA strand of a given locus to efficiently bind and process it. Such novel configuration thus extends the range of TALE nucleases applications.

Today, the conventional TALE nuclease architecture used by most investigators results from the symmetrical association of two TALE::FokI scaffolds (Figure 1A and 1D). To be active as nuclease entity, this scaffolds need to bind in a Tail to Tail (TtT) orientation to adjacent binding sites bearing a T0 at their 5’ ends and located on the reverse and forward strand of the locus to process. Although highly efficient, such architecture is unable to process loci devoided of thymidine residues either on one or two strands. To overcome such requirement for T0 and at the same time, to extend the range of TALE-based applications, two different groups employed rational design and directed evolution to modify the TALE N-terminal domain, a region reported to play a key role in the T0 specificity of TALE [31,32]. In another study, such requirement for T0 was reported to be overcome by using a new modular base-per-base binding domains (M3BD) scaffold from *Burkholderia rhizoxinica*[33].Relaxing the T0 specificity of TALE binding domain might not be the only strategy to extend the range of TALE-based applications. Alternative approaches, exploiting the versatility of the TALE scaffold, could also be considered to abrogate the requirement of two T0 located on adjacent and anti-parallel TALE binding sites while still delivering efficient TALE nuclease activity. To develop such alternative approach, we first ruled out altering the T0 specificity of the TALE N-terminal domain, considering that keeping this binding anchor at the 5’ end of each TALE DNA binding domain would be beneficial for the specificity and safety of the resulting designer nuclease. We thus sought to set up a novel dimeric TALE nuclease architecture that would require two adjacent TALE binding sites located on only one strand of the locus of interest. Because of the oriented fashion of TALE domain binding and the requirement for FokI dimerization, such architecture named TtH, required the asymmetrical association of two different TALE scaffolds, the TALE::FokI and the FokI::TALE in a Tail to Head orientation (Figure 1E).

We developed and tested combinations of the FokI::TALE and TALE::FokI scaffolds and found that the symmetrical HtH and TtT architectures as well as the asymmetrical TtH architecture could efficiently catalyze targeted DSBs in a yeast-based nuclease assay. Although the different architectures displayed similar activity levels, they showed marked differences regarding the properties of their targets. Interestingly, the narrower spacer range cleaved by the two new HtH and TtH architectures could represent an advantage by reducing the number of potential off-site targets in a genome of interest. Indeed, off-site targets are usually determined as sequences (combinations of left + right, left + left, right + right sequences) diverging from the intended target site by a few base pairs. In addition, to be considered as a potential off-site target, the two binding sites have to be separated by a spacer compatible with a nuclease activity. Based on the results presented in this study (Figure 1D), 25 different spacers have to be taken in account for the “classical” TtT architecture. Regarding the HtH and TtH architectures only 8 or 16 different spacer lengths need to be considered respectively (Figures 1C and E). These differences resulted in an approximate 3 and 2 fold higher numbers of potential off-site targets for the TtT compared to the HtH and TtH architectures respectively. In addition, we believed that the global nuclease activity and specificity could still be improved by optimizing the flexibility and/or rigidity of the aminoacid linker between the FokI and TALE domain. Indeed, Mercer and colleagues have recently shown that fusion of recombinase catalytic domain to alternative truncations of TALE N-terminal domain (Δ120 or Δ128) could enhance the efficiency of their chimeric TALE recombinase system [12].

As the levels of activities obtained in the yeast SSA assay were fully satisfactory, we evaluated the performance of the two HtH and TtH architectures in a chromosomal context. We thus generated three pairs of nucleases for both architectures following the guidelines (spacer length) obtained previously in yeast, to target a total of six loci in two mammalian cell types (CHO-KI and 293H). The efficiency of these nucleases to induce DSB events was monitored 3 days post transfection by measuring Indels generated by NHEJ at their cleavage sites. We cannot exclude that variation in nuclease activity is dependent on multiple parameters and is thus not exclusively resulting from the difference in architecture. Indeed, the RVD composition of the nuclease, the targeted DNA sequence and the presence of micro-homologies within the targeted loci are likely to influence several biochemical parameters such as overall DNA binding, cleavage efficiency and global DNA repair outcome. Nevertheless, we found that both architectures induced a substantial level of targeted mutagenesis (Table 1). Additionally, the molecular characterization of deletion events allowed us to observe that the DSB occurred in the middle of the spacer region for the TtT and HtH architecture while being shifted toward the TALE::FokI scaffold binding site of the new TtH architecture.

While TtH architecture does not significantly increase the number of targetable loci, it could be endowed with a new technological advantage for the field of gene therapy. Indeed, when considering the human chromosome 1 as a model, and using standard criteria for the array (15.5 repeats) and spacer (10-16 and 20-25 bp) length [26], we estimated that the classical TtT architecture could target about 99.8% of this chromosome. The remaining 0.2% comprised a total of 2945 regions of 68 to 1983 bp (representing a total of 346422 nucleotides) that were devoided of any TtT nuclease target. We noted that more than half of these sequences could be potentially targeted using the TtH configuration (array of 15.5 repeats and spacer of 17-23 and 26-33 bp, Figure 1E) and that an important proportion of these sequences was composed of highly repetitive motives. Expansion of triplets or quadruplets is commonly linked to several genetic disorders and different neurological syndromes [34]. Their expansion induces aberrant protein expressions and subsequent aggregation, as well as formation and persistence of RNA:DNA hybrids responsible for genomic instability and inhibition of replication. Processing such pathogenic sequences represents important therapeutic potentials to cure their related genetic disease and, in that matter, the advantages of the new TtH architecture are twofold with respect to conventional TALEN architecture. First, it could process and thus stimulate contraction of expanded sequences harboring thymidine on one unique strand (CAG, GAA, CTG and CCTG). Second, through the well known ability of FokI to cleave DNA:RNA hybrids along with the capacity of TALE domain to interact with such molecule [35], it could also reduce the deleterious downstream effects of DNA:RNA duplex via their targeted processing. Noteworthy, such approach could also be used in general to process DNA:RNA hybrids.

Finally, besides its potential for specific gene therapy application, the TtH architecture could be beneficial for the field of genome editing by allowing for more precise positioning of TALE nuclease without affecting their T0 specificity, one of the hallmark of their DNA specificity.

## Conclusions

Overall, although a larger dataset would be desirable, our results demonstrate that the level of genome modifications that can be obtained in mammalian cells with the new TtH architecture is compatible with most, if not all, genome editing applications. An additional benefit of this particular asymmetrical architecture is the availability of both N- and C-termini that can also expand the “cargo” possibility of TALE-based nucleases. We believe that this particular TtH architecture will further expand the possibilities of the TALE-based nuclease technology for targeting sequences with biased nucleotide composition (e.g. highly repetitive motives) or DNA-RNA hybrids.

## Methods

### TALE arrays

All TALE arrays were obtained from Cellectis Bioresearch (Paris, France). TALEN™ is a trademark owned by Cellectis Bioresearch. Sequences of TALE-nuclease backbones, TALE RVD array composition and/or relevant targets are presented in the Additional file 1. For experiments in mammalian cell lines, the TALE-based nucleases were expressed under the control of either an EF1a promoter (HtH and TtH architectures) or a CMV promoter (TtT architecture).

### Extrachromosomal SSA assay in yeast

TALE-based nuclease containing yeast strain were gridded at high gridding density (~20 spots/cm^2^) on nylon filters placed on solid agar containing YP-glycerol plates, using a colony gridder (QpixII, Genetix). A second layer, consisting of reporter-harboring yeast strains, was gridded on the same filter for each target. Membranes were incubated overnight at 30°C to allow mating. To select for diploids, filters were then places and incubated for 2 days at 30°C on medium containing glucose (2%) as the carbon source but lacking leucine (for the TALE nuclease left arm), tryptophan (for the target) and supplemented with G418 (for the TALE nuclease right arm, if required). To induce the expression of the TALE-based nuclease, filters were transferred onto YP-galactose-rich medium for 24-48 hours at 30°C or 37°C. To monitor nuclease activity, through the β-galactosidase activity, filters were finally placed on solid agarose medium containing 0.02% X-Gal in 0.5 M sodium phosphate buffer, pH 7.0, 0.1% SDS, 6% dimethyl formamide (DMF), 7 mM β-mercaptoethanol, 1% agarose and incubated at 37°C for up to 48 h.

Filters were scanned and each spot was quantified using the median values of the pixels constituting the spot. We attribute the arbitrary values 0 and 1 to white and dark pixels, respectively. β-Galactosidase activity is directly associated with the efficiency of homologous recombination, thus with the cleavage efficiency of the TALE-based nuclease. Any value >0 is considered as the consequence of cleavage.

### Nuclease transfection in 293H cells

Human 293H cells (Life Technologies) were cultured at 37°C with 5% CO_2_ in DMEM complete medium supplemented with 2 mM L-glutamine, penicillin (100 IU/ml), streptomycin (100 μg/ml), amphotericin B (Fongizone: 0.25 μg/ml, Life Technologies,) and 10% FBS. Adherent 293H cells were seeded at 1.2 10^6^ cells in 10 cm Petri dishes one day before transfection. Cell transfection was performed using the Lipofectamine 2000 reagent according to the manufacturer’s instructions (Invitrogen). In brief, 2.5 μg (for the HtH and TtH architectures) or 12 μg (for the TtT architecture) of each of the two nuclease expression vector pairs, and 10 ng of GFP expression vector (to monitor transfection efficiencies) were mixed with 0.3 ml of DMEM without FBS (5 μg final DNA amount). In another tube 25 μL of Lipofectamine were mixed with 0.3 ml of DMEM without FBS. After 5 minutes incubation, both DNA and Lipofectamine mixes were combined and incubated for 20 min at RT. The mixture was transferred to a Petri dish containing the 293H cells in 9 ml of complete medium and then cultured at 37°C under 5% CO_2_. Three days post-transfection, the cells were washed with phosphate-buffered saline (PBS), trypsinized, resuspended in 5 ml complete medium and the percentage of GFP positive cells was measured by flow cytometry (Guava EasyCyte) in order to monitor transfection efficacy.

### Nuclease transfection in CHO-KI cells

CHO-K1 cells (ATCC) were cultured at 37°C with 5% CO_2_ in F-12 K complete medium (Gibco) supplemented with 2 mM L-glutamine, penicillin (100 IU/ml), streptomycin (100 μg/ml), amphotericin B (Fongizone: 0.25 μg/ml, Life Technologies,) and 10% FBS. Cell transfection was performed by electroporation with the Nucleofector Kit T for CHO-K1 cells (Lonza) according to the manufacturer’s protocol. Cells (1 x 10^6^ cells) were transfected with 5 μg of the two nuclease expression vector pairs and 10 ng of GFP expression vector (to monitor transfection efficiencies) (10 μg final DNA amount), then plated in a 10 cm dish in complete medium (F-12 K medium, Gibco) supplemented with 2 mM L-glutamine, penicillin (100 IU/ml), streptomycin (100 μg/ml), Fongizone (0.25 μg/ml) and 10% FBS. Three days post-transfection, the cells were washed with phosphate-buffered saline (PBS), trypsinized, resuspended in 5 ml complete medium and the percentage of GFP positive cells was measured by flow cytometry (Guava EasyCyte) in order to monitor transfection efficacy.

### Targeted mutagenesis

Cells were pelleted by centrifugation and genomic DNA was extracted using DNeasy Blood & Tissue Kit (Qiagen) according to the manufacturer’s instructions. PCR of the endogenous locus (370-630 bp final product size) were performed using the oligonucleotide sequences presented in the Additional file 1 and purified using the AMPure kit (Invitrogen). Amplicons were further analyzed by deep sequencing using the 454 system (Roche) [36].

### Ethics

The research study described in the manuscript does not involve human subjects, human material, or human data. All the cell lines were purchased from ATCC (Virginia, USA)

## Competing interests

All co-authors are present or former Cellectis employees.

## Authors’ contributions

AJ, MB, JV and PD conceived the study and designed experiments. AJ, MB, JV, ST and GD performed experiments. FB, GHS, FD and AD provided conceptual and technical advices. AJ, MB, JV, MZ, and JM analyzed experiments. AJ, MB, JV and PD wrote the manuscript with support from all authors. All authors read and approved the final manuscript.

## Supplementary Material

Additional file 1 Additional Data. Click here for file
