# Supplementary material for: Exploring the transcription activator-like effectors scaffold versatility to expand the toolbox of designer nucleases
Source: BMC Mol Biol. 2014 Jul 5;15:13. doi: 10.1186/1471-2199-15-13 (PMC4099384; doi:10.1186/1471-2199-15-13)
Supplement: Additional file 1 — Additional Data. [file 1471-2199-15-13-S1.docx]

**Additional Data**

**Exploring the transcription activator-like effectors scaffold versatility to expand the toolbox of designer nucleases**

**Alexandre Juillerat^1^**†**^*^, Marine Beurdeley^1^**†**, Julien Valton^1^**†**, Séverine Thomas^1^, Gwendoline Dubois^1^, Mikhail Zaslavskiy^1^, Jérome Mikolajczak^1^, Fabian Bietz^1^, George H. Silva^1^, Aymeric Duclert^1^, Fayza Daboussi^1^ and Philippe Duchateau^1*^**

* Corresponding author: Philippe Duchateau [philippe.duchateau@cellectis.com](mailto:philippe.duchateau@cellectis.com) – Alexandre Juillerat [alexandre.juillerat@cellectis.com](mailto:alexandre.juillerat@cellectis.com)

† Equal contributors

CELLECTIS S.A., 8 Rue de la Croix Jarry, 75013 Paris, France.

**Supplementary table 1**

Amino acid sequences of N-terminal and C-terminal (including FokI) domains used in the used in the N-terminal FokI fusions (yeast experiments).

| **Nter Sequence (with FokI)** | MASGPNRGVTKQLVKSELEEKKSELRHKLKYVPHEYIELIEIARNSTQDRILEMKVMEFFMKVYGYRGKHLGGSRKPDGAIYTVGSPIDYGVIVDTKAYSGGYNLPIGQADEMQRYVEENQTRNKHINPNEWWKVYPSSVTEFKFLFVSGHFKGNYKAQLTRLNHITNCNGAVLSVEELLIGGEMIKAGTLTLEEVRRKFNNGEINFGSSGVDLRTLGYSQQQQEKIKPKVRSTVAQHHEALVGHGFTHAHIVALSQHPAALGTVAVKYQDMIAALPEATHEAIVGVGKQWSGARALEALLTVAGELRGPPLQLDTGQLLKIAKRGGVTAVEAVHAWRNALTGAPLN |
| --- | --- |
| **Cter Sequence** | SIVAQLSRPDPSAD |

Amino acid sequences of N-terminal and C-terminal (including FokI) domains used in the used in the C-terminal FokI fusions (yeast experiments).

| **Nter Sequence** | MADPIRSRTPSPARELLPGPQPDGVQPTADRGVSPPAGGPLDGLPARRTMSRTRLPSPPAPSPAFSAGSFSDLLRQFDPSLFNTSLFDSLPPFGAHHTEAATGEWDEVQSGLRAADAPPPTMRVAVTAARPPRAKPAPRRRAAQPSDASPAAQVDLRTLGYSQQQQEKIKPKVRSTVAQHHEALVGHGFTHAHIVALSQHPAALGTVAVKYQDMIAALPEATHEAIVGVGKQWSGARALEALLTVAGELRGPPLQLDTGQLLKIAKRGGVTAVEAVHAWRNALTGAPLN |
| --- | --- |
| **Cter Sequence (with FokI)** | SIVAQLSRPDPALAALTNDHLVALACLGGRPALDAVKKGLGDPISRSQLVKSELEEKKSELRHKLKYVPHEYIELIEIARNSTQDRILEMKVMEFFMKVYGYRGKHLGGSRKPDGAIYTVGSPIDYGVIVDTKAYSGGYNLPIGQADEMQRYVEENQTRNKHINPNEWWKVYPSSVTEFKFLFVSGHFKGNYKAQLTRLNHITNCNGAVLSVEELLIGGEMIKAGTLTLEEVRRKFNNGEINFAAD |

TALEN array composition used in the Tail to Tail (TtT) and Head to Head (HtH) architectures preliminary experiments.

| **FokI fusion** | **RVD Array sequence** |
| --- | --- |
| N-ter or C-ter | [HD-NG-NI-NG-NI-NI-NI-HD-HD-NG-NI-NI-HD-HD-HD-NG-HD-NG#](http://bioinfo.cellectis.com/lims/st2fr/sequence/sequenceManagerQuery.action?multipleNames=&multipleRvdMotifs=HD-NG-NI-NG-NI-NI-NI-HD-HD-NG-NI-NI-HD-HD-HD-NG-HD-NG%23) |

TALEN arrays compositions used in the Tail to Head (TtH) architecture preliminary experiments.

| **FokI fusion** | **RVD Array sequence** |
| --- | --- |
| N-ter | [HD-NG-NI-NG-NI-NI-NI-HD-HD-NG-NI-NI-HD-HD-HD-NG-HD-NG#](http://bioinfo.cellectis.com/lims/st2fr/sequence/sequenceManagerQuery.action?multipleNames=&multipleRvdMotifs=HD-NG-NI-NG-NI-NI-NI-HD-HD-NG-NI-NI-HD-HD-HD-NG-HD-NG%23) |
| C-ter | [NN-NG-NG-NG-NI-NG-NN-NN-NG-NG-NI-HD-NG-NG-NI-NG#](http://bioinfo.cellectis.com/lims/st2fr/sequence/sequenceManagerQuery.action?multipleNames=&multipleRvdMotifs=NN-NG-NG-NG-NI-NG-NN-NN-NG-NG-NI-HD-NG-NG-NI-NG%23) |

**Supplementary table 2**

Target sequences (upper case) with spacer (lower case) length ranging from 5 to 35 base pairs for the Head to Head (HtH) architecture.

| **Spacer size** | **Target sequence** |
| --- | --- |
| **5** | AGAGGGTTAGGTTTATATAaggtaTATATAAACCTAACCCTCT |
| **6** | AGAGGGTTAGGTTTATATAaaggtaTATATAAACCTAACCCTCT |
| **7** | AGAGGGTTAGGTTTATATAaaggtacTATATAAACCTAACCCTCT |
| **8** | AGAGGGTTAGGTTTATATAgaaggtacTATATAAACCTAACCCTCT |
| **9** | AGAGGGTTAGGTTTATATAgaaggtaccTATATAAACCTAACCCTCT |
| **10** | AGAGGGTTAGGTTTATATAtgaaggtaccTATATAAACCTAACCCTCT |
| **11** | AGAGGGTTAGGTTTATATAtgaaggtacctTATATAAACCTAACCCTCT |
| **12** | AGAGGGTTAGGTTTATATAatgaaggtacctTATATAAACCTAACCCTCT |
| **13** | AGAGGGTTAGGTTTATATAatgaaggtaccttTATATAAACCTAACCCTCT |
| **14** | AGAGGGTTAGGTTTATATAcatgaaggtaccttTATATAAACCTAACCCTCT |
| **15** | AGAGGGTTAGGTTTATATAcatgaaggtaccttgTATATAAACCTAACCCTCT |
| **16** | AGAGGGTTAGGTTTATATAgcatgaaggtaccttgTATATAAACCTAACCCTCT |
| **17** | AGAGGGTTAGGTTTATATAgcatgaaggtaccttgtTATATAAACCTAACCCTCT |
| **18** | AGAGGGTTAGGTTTATATAagcatgaaggtaccttgtTATATAAACCTAACCCTCT |
| **19** | AGAGGGTTAGGTTTATATAagcatgaaggtaccttgtcTATATAAACCTAACCCTCT |
| **20** | AGAGGGTTAGGTTTATATAtagcatgaaggtaccttgtcTATATAAACCTAACCCTCT |
| **21** | AGAGGGTTAGGTTTATATAtagcatgaaggtaccttgtcgTATATAAACCTAACCCTCT |
| **22** | AGAGGGTTAGGTTTATATAtagcatgaaggtaccttgtcgtTATATAAACCTAACCCTCT |
| **23** | AGAGGGTTAGGTTTATATActagcatgaaggtaccttgtcgtTATATAAACCTAACCCTCT |
| **24** | AGAGGGTTAGGTTTATATActagcatgaaggtaccttgtcgttTATATAAACCTAACCCTCT |
| **25** | AGAGGGTTAGGTTTATATAactagcatgaaggtaccttgtcgttTATATAAACCTAACCCTCT |
| **26** | AGAGGGTTAGGTTTATATAactagcatgaaggtaccttgtcgttgTATATAAACCTAACCCTCT |
| **27** | AGAGGGTTAGGTTTATATAcactagcatgaaggtaccttgtcgttgTATATAAACCTAACCCTCT |
| **28** | AGAGGGTTAGGTTTATATAcactagcatgaaggtaccttgtcgttgaTATATAAACCTAACCCTCT |
| **29** | AGAGGGTTAGGTTTATATAccactagcatgaaggtaccttgtcgttgaTATATAAACCTAACCCTCT |
| **30** | AGAGGGTTAGGTTTATATAccactagcatgaaggtaccttgtcgttgatTATATAAACCTAACCCTCT |
| **31** | AGAGGGTTAGGTTTATATAaccactagcatgaaggtaccttgtcgttgatTATATAAACCTAACCCTCT |
| **32** | AGAGGGTTAGGTTTATATAaccactagcatgaaggtaccttgtcgttgattTATATAAACCTAACCCTCT |
| **33** | AGAGGGTTAGGTTTATATAgaccactagcatgaaggtaccttgtcgttgattTATATAAACCTAACCCTCT |
| **34** | AGAGGGTTAGGTTTATATAgaccactagcatgaaggtaccttgtcgttgattcTATATAAACCTAACCCTCT |
| **35** | AGAGGGTTAGGTTTATATAtgaccactagcatgaaggtaccttgtcgttgattcTATATAAACCTAACCCTCT |

**Supplementary table 3**

Target sequences (upper case) with spacer (lower case) length ranging from 5 to 35 base pairs for the Tail to Tail (TtT) architecture.

| **Spacer size** | **Target sequence** |
| --- | --- |
| **5** | TATATAAACCTAACCCTCTaggtaAGAGGGTTAGGTTTATATA |
| **6** | TATATAAACCTAACCCTCTaaggtaAGAGGGTTAGGTTTATATA |
| **7** | TATATAAACCTAACCCTCTaaggtacAGAGGGTTAGGTTTATATA |
| **8** | TATATAAACCTAACCCTCTgaaggtacAGAGGGTTAGGTTTATATA |
| **9** | TATATAAACCTAACCCTCTgaaggtaccAGAGGGTTAGGTTTATATA |
| **10** | TATATAAACCTAACCCTCTtgaaggtaccAGAGGGTTAGGTTTATATA |
| **11** | TATATAAACCTAACCCTCTtgaaggtacctAGAGGGTTAGGTTTATATA |
| **12** | TATATAAACCTAACCCTCTatgaaggtacctAGAGGGTTAGGTTTATATA |
| **13** | TATATAAACCTAACCCTCTatgaaggtaccttAGAGGGTTAGGTTTATATA |
| **14** | TATATAAACCTAACCCTCTcatgaaggtaccttAGAGGGTTAGGTTTATATA |
| **15** | TATATAAACCTAACCCTCTtagcatgaaggtaccAGAGGGTTAGGTTTATATA |
| **16** | TATATAAACCTAACCCTCTgcatgaaggtaccttgAGAGGGTTAGGTTTATATA |
| **17** | TATATAAACCTAACCCTCTgcatgaaggtaccttgtAGAGGGTTAGGTTTATATA |
| **18** | TATATAAACCTAACCCTCTagcatgaaggtaccttgtAGAGGGTTAGGTTTATATA |
| **19** | TATATAAACCTAACCCTCTagcatgaaggtaccttgtcAGAGGGTTAGGTTTATATA |
| **20** | TATATAAACCTAACCCTCTtagcatgaaggtaccttgtcAGAGGGTTAGGTTTATATA |
| **21** | TATATAAACCTAACCCTCTtagcatgaaggtaccttgtcgAGAGGGTTAGGTTTATATA |
| **22** | TATATAAACCTAACCCTCTtagcatgaaggtaccttgtcgtAGAGGGTTAGGTTTATATA |
| **23** | TATATAAACCTAACCCTCTctagcatgaaggtaccttgtcgtAGAGGGTTAGGTTTATATA |
| **24** | TATATAAACCTAACCCTCTctagcatgaaggtaccttgtcgttAGAGGGTTAGGTTTATATA |
| **25** | TATATAAACCTAACCCTCTactagcatgaaggtaccttgtcgttAGAGGGTTAGGTTTATATA |
| **26** | TATATAAACCTAACCCTCTactagcatgaaggtaccttgtcgttgAGAGGGTTAGGTTTATATA |
| **27** | TATATAAACCTAACCCTCTcactagcatgaaggtaccttgtcgttgAGAGGGTTAGGTTTATATA |
| **28** | TATATAAACCTAACCCTCTcactagcatgaaggtaccttgtcgttgaAGAGGGTTAGGTTTATATA |
| **29** | TATATAAACCTAACCCTCTccactagcatgaaggtaccttgtcgttgaAGAGGGTTAGGTTTATATA |
| **30** | TATATAAACCTAACCCTCTccactagcatgaaggtaccttgtcgttgatAGAGGGTTAGGTTTATATA |
| **31** | TATATAAACCTAACCCTCTaccactagcatgaaggtaccttgtcgttgatAGAGGGTTAGGTTTATATA |
| **32** | TATATAAACCTAACCCTCTaccactagcatgaaggtaccttgtcgttgattAGAGGGTTAGGTTTATATA |
| **33** | TATATAAACCTAACCCTCTgaccactagcatgaaggtaccttgtcgttgattAGAGGGTTAGGTTTATATA |
| **34** | TATATAAACCTAACCCTCTgaccactagcatgaaggtaccttgtcgttgattcAGAGGGTTAGGTTTATATA |
| **35** | TATATAAACCTAACCCTCTtgaccactagcatgaaggtaccttgtcgttgattcAGAGGGTTAGGTTTATATA |

**Supplementary table 4**

Target sequences (upper case) with spacer (lower case) length ranging from 5 to 35 base pairs for the Tail to Head (TtH) architecture.

| **Spacer size** | **Target sequence** |
| --- | --- |
| **5** | TGTTTATGGTTACTTATaggtaTATATAAACCTAACCCTCT |
| **6** | TGTTTATGGTTACTTATaaggtaTATATAAACCTAACCCTCT |
| **7** | TGTTTATGGTTACTTATaaggtacTATATAAACCTAACCCTCT |
| **8** | TGTTTATGGTTACTTATgaaggtacTATATAAACCTAACCCTCT |
| **9** | TGTTTATGGTTACTTATgaaggtaccTATATAAACCTAACCCTCT |
| **10** | TGTTTATGGTTACTTATtgaaggtaccTATATAAACCTAACCCTCT |
| **11** | TGTTTATGGTTACTTATtgaaggtacctTATATAAACCTAACCCTCT |
| **12** | TGTTTATGGTTACTTATatgaaggtacctTATATAAACCTAACCCTCT |
| **13** | TGTTTATGGTTACTTATatgaaggtaccttTATATAAACCTAACCCTCT |
| **14** | TGTTTATGGTTACTTATcatgaaggtaccttTATATAAACCTAACCCTCT |
| **15** | TGTTTATGGTTACTTATtagcatgaaggtaccTATATAAACCTAACCCTCT |
| **16** | TGTTTATGGTTACTTATgcatgaaggtaccttgTATATAAACCTAACCCTCT |
| **17** | TGTTTATGGTTACTTATgcatgaaggtaccttgtTATATAAACCTAACCCTCT |
| **18** | TGTTTATGGTTACTTATagcatgaaggtaccttgtTATATAAACCTAACCCTCT |
| **19** | TGTTTATGGTTACTTATagcatgaaggtaccttgtcTATATAAACCTAACCCTCT |
| **20** | TGTTTATGGTTACTTATtagcatgaaggtaccttgtcTATATAAACCTAACCCTCT |
| **21** | TGTTTATGGTTACTTATtagcatgaaggtaccttgtcgTATATAAACCTAACCCTCT |
| **22** | TGTTTATGGTTACTTATtagcatgaaggtaccttgtcgtTATATAAACCTAACCCTCT |
| **23** | TGTTTATGGTTACTTATctagcatgaaggtaccttgtcgtTATATAAACCTAACCCTCT |
| **24** | TGTTTATGGTTACTTATctagcatgaaggtaccttgtcgttTATATAAACCTAACCCTCT |
| **25** | TGTTTATGGTTACTTATactagcatgaaggtaccttgtcgttTATATAAACCTAACCCTCT |
| **26** | TGTTTATGGTTACTTATactagcatgaaggtaccttgtcgttgTATATAAACCTAACCCTCT |
| **27** | TGTTTATGGTTACTTATcactagcatgaaggtaccttgtcgttgTATATAAACCTAACCCTCT |
| **28** | TGTTTATGGTTACTTATcactagcatgaaggtaccttgtcgttgaTATATAAACCTAACCCTCT |
| **29** | TGTTTATGGTTACTTATccactagcatgaaggtaccttgtcgttgaTATATAAACCTAACCCTCT |
| **30** | TGTTTATGGTTACTTATccactagcatgaaggtaccttgtcgttgatTATATAAACCTAACCCTCT |
| **31** | TGTTTATGGTTACTTATaccactagcatgaaggtaccttgtcgttgatTATATAAACCTAACCCTCT |
| **32** | TGTTTATGGTTACTTATaccactagcatgaaggtaccttgtcgttgattTATATAAACCTAACCCTCT |
| **33** | TGTTTATGGTTACTTATgaccactagcatgaaggtaccttgtcgttgattTATATAAACCTAACCCTCT |
| **34** | TGTTTATGGTTACTTATgaccactagcatgaaggtaccttgtcgttgattcTATATAAACCTAACCCTCT |
| **35** | TGTTTATGGTTACTTATtgaccactagcatgaaggtaccttgtcgttgattcTATATAAACCTAACCCTCT |

**Supplementary table 5**

Amino acid sequences of N-terminal and C-terminal (including FokI) domains used in the used in the N-terminal FokI fusions (mammalian experiments).

| **Nter Sequence (with FokI)** | MASPKKKRKVEGNSKETAAAKFERQHMDSGSADMASGPNRGVTKQLVKSELEEKKSELRHKLKYVPHEYIELIEIARNSTQDRILEMKVMEFFMKVYGYRGKHLGGSRKPDGAIYTVGSPIDYGVIVDTKAYSGGYNLPIGQADEMQRYVEENQTRNKHINPNEWWKVYPSSVTEFKFLFVSGHFKGNYKAQLTRLNHITNCNGAVLSVEELLIGGEMIKAGTLTLEEVRRKFNNGEINFGSSGVDLRTLGYSQQQQEKIKPKVRSTVAQHHEALVGHGFTHAHIVALSQHPAALGTVAVKYQDMIAALPEATHEAIVGVGKQWSGARALEALLTVAGELRGPPLQLDTGQLLKIAKRGGVTAVEAVHAWRNALTGAPLN |
| --- | --- |
| **Cter Sequence** | SIVAQLSRPDPSAD |

Amino acid sequences of N-terminal and C-terminal (including FokI) domains used in the used in the C-terminal FokI fusions (mammalian experiments).

| **Nter Sequence** | MGDPKKKRKVIDYPYDVPDYAIDIADPIRSRTPSPARELLPGPQPDGVQPTADRGVSPPAGGPLDGLPARRTMSRTRLPSPPAPSPAFSAGSFSDLLRQFDPSLFNTSLFDSLPPFGAHHTEAATGEWDEVQSGLRAADAPPPTMRVAVTAARPPRAKPAPRRRAAQPSDASPAAQVDLRTLGYSQQQQEKIKPKVRSTVAQHHEALVGHGFTHAHIVALSQHPAALGTVAVKYQDMIAALPEATHEAIVGVGKQWSGARALEALLTVAGELRGPPLQLDTGQLLKIAKRGGVTAVEAVHAWRNALTGAPLN |
| --- | --- |
| **Cter Sequence (with FokI)** | SIVAQLSRPDPALAALTNDHLVALACLGGRPALDAVKKGLGDPISRSQLVKSELEEKKSELRHKLKYVPHEYIELIEIARNSTQDRILEMKVMEFFMKVYGYRGKHLGGSRKPDGAIYTVGSPIDYGVIVDTKAYSGGYNLPIGQADEMQRYVEENQTRNKHINPNEWWKVYPSSVTEFKFLFVSGHFKGNYKAQLTRLNHITNCNGAVLSVEELLIGGEMIKAGTLTLEEVRRKFNNGEINFAAD |

TALEN array RVD composition and target sequences used for the Head to Head (HtH) architectures.

| **Locus** | **RVD Array compositions** | **Target Sequence** |
| --- | --- | --- |
| **RAG1** | [NG-HD-HD-NI-HD-NI-NI-NI-NI-NI-HD-HD-NG-NG-NI-NG#](http://bioinfo.cellectis.com/lims/st2fr/sequence/sequenceManagerQuery.action?multipleNames=&multipleRvdMotifs=NG-HD-HD-NI-HD-NI-NI-NI-NI-NI-HD-HD-NG-NG-NI-NG%23)  [NG-NG-NI-NI-NN-HD-NI-HD-NG-NG-NI-NG-NI-NG-NN-NG#](http://bioinfo.cellectis.com/lims/st2fr/sequence/sequenceManagerQuery.action?multipleNames=&multipleRvdMotifs=NG-NG-NI-NI-NN-HD-NI-HD-NG-NG-NI-NG-NI-NG-NN-NG%23) | TCCACAAAAACCTTAA  TTAAGCACTTATATGT |
| **DMD** | [HD-HD-NI-NG-NI-NI-NI-NN-NN-NI-NI-NG-NI-HD-NI-NG#](http://bioinfo.cellectis.com/lims/st2fr/sequence/sequenceManagerQuery.action?multipleNames=&multipleRvdMotifs=HD-HD-NI-NG-NI-NI-NI-NN-NN-NI-NI-NG-NI-HD-NI-NG%23)  [NG-NI-NN-HD-NG-HD-NI-NG-NG-NG-HD-NG-HD-NG-NI-NG#](http://bioinfo.cellectis.com/lims/st2fr/sequence/sequenceManagerQuery.action?multipleNames=&multipleRvdMotifs=NG-NI-NN-HD-NG-HD-NI-NG-NG-NG-HD-NG-HD-NG-NI-NG%23) | CCATAAAGGAATACAG  TAGCTCATTTCTCTAA |
| **FUT8** | [NG-NN-NI-HD-NI-NG-HD-HD-NI-NG-NI-NN-HD-HD-NI-NG#](http://bioinfo.cellectis.com/lims/st2fr/sequence/sequenceManagerQuery.action?multipleNames=&multipleRvdMotifs=NG-NN-NI-HD-NI-NG-HD-HD-NI-NG-NI-NN-HD-HD-NI-NG%23)  [NN-NI-NG-NG-NN-HD-NG-NG-NI-NG-NN-NN-HD-NI-HD-NG#](http://bioinfo.cellectis.com/lims/st2fr/sequence/sequenceManagerQuery.action?multipleNames=&multipleRvdMotifs=NN-NI-NG-NG-NN-HD-NG-NG-NI-NG-NN-NN-HD-NI-HD-NG%23) | TGACATCCATAGCCAC  GATTGCTTATGGCACC |

TALEN array RVD composition and target sequences used for the Tail to Head (TtH) architectures.

| **Locus** | **RVD Array compositions** | **Target Sequence** |
| --- | --- | --- |
| **RAG1** | [NG-NI-NG-NI-NG-NN-NG-NN-NG-NN-NG-NI-NI-HD-NI-NG#](http://bioinfo.cellectis.com/lims/st2fr/sequence/sequenceManagerQuery.action?multipleNames=&multipleRvdMotifs=NG-NI-NG-NI-NG-NN-NG-NN-NG-NN-NG-NI-NI-HD-NI-NG%23)  [NN-NG-HD-NI-NN-NI-NI-NN-NI-NN-NN-NI-NI-NI-NG-NG#](http://bioinfo.cellectis.com/lims/st2fr/sequence/sequenceManagerQuery.action?multipleNames=&multipleRvdMotifs=NN-NG-HD-NI-NN-NI-NI-NN-NI-NN-NN-NI-NI-NI-NG-NG%23) | TATATGTGTGTAACAG  GTCAGAAGAGGAAATA |
| **DMD** | [NN-HD-NG-NN-NG-NG-NI-NG-HD-NG-HD-NI-NN-NG-HD-NG#](http://bioinfo.cellectis.com/lims/st2fr/sequence/sequenceManagerQuery.action?multipleNames=&multipleRvdMotifs=NN-HD-NG-NN-NG-NG-NI-NG-HD-NG-HD-NI-NN-NG-HD-NG%23)  [NG-NG-NI-NG-NN-NN-NI-NG-HD-NI-NN-NG-NG-NI-NI-NG#](http://bioinfo.cellectis.com/lims/st2fr/sequence/sequenceManagerQuery.action?multipleNames=&multipleRvdMotifs=NG-NG-NI-NG-NN-NN-NI-NG-HD-NI-NN-NG-NG-NI-NI-NG%23) | GCTGTTATCTCAGTCA  TTATGGATCAGTTAAC |
| **FUT8** | [NG-HD-NI-NG-NN-NI-NG-NG-NN-HD-NG-NG-NI-NG-NN-NG#](http://bioinfo.cellectis.com/lims/st2fr/sequence/sequenceManagerQuery.action?multipleNames=&multipleRvdMotifs=NG-HD-NI-NG-NN-NI-NG-NG-NN-HD-NG-NG-NI-NG-NN-NG%23)  [NN-NN-NI-NI-NG-HD-NG-HD-NI-NN-NI-NI-NG-NG-NN-NG#](http://bioinfo.cellectis.com/lims/st2fr/sequence/sequenceManagerQuery.action?multipleNames=&multipleRvdMotifs=NN-NN-NI-NI-NG-HD-NG-HD-NI-NN-NI-NI-NG-NG-NN-NG%23) | TCATGATTGCTTATGG  GGAATCTCAGAATTGG |

**Supplementary table 6**

Amino acid sequences of N-terminal and C-terminal (including FokI) domains used for the TtT architecture (mammalian experiments).

Monomer 1

| **Nter Sequence** | MGDPKKKRKVIDYPYDVPDYAIDIADLRTLGYSQQQQEKIKPKVRSTVAQHHEALVGHGFTHAHIVALSQHPAALGTVAVKYQDMIAALPEATHEAIVGVGKQWSGARALEALLTVAGELRGPPLQLDTGQLLKIAKRGGVTAVEAVHAWRNALTGAPLN |
| --- | --- |
| **Cter Sequence (with FokI)** | SIVAQLSRPDPALAALTNDHLVALACLGGRPALDAVKKGLGDPISRSQLVKSELEEKKSELRHKLKYVPHEYIELIEIARNSTQDRILEMKVMEFFMKVYGYRGKHLGGSRKPDGAIYTVGSPIDYGVIVDTKAYSGGYNLPIGQADEMQRYVEENQTRNKHINPNEWWKVYPSSVTEFKFLFVSGHFKGNYKAQLTRLNHITNCNGAVLSVEELLIGGEMIKAGTLTLEEVRRKFNNGEINFAAA |

Monomer 2

| **Nter Sequence** | MGDPKKKRKVIDKETAAAKFERQHMDSIDIADLRTLGYSQQQQEKIKPKVRSTVAQHHEALVGHGFTHAHIVALSQHPAALGTVAVKYQDMIAALPEATHEAIVGVGKQWSGARALEALLTVAGELRGPPLQLDTGQLLKIAKRGGVTAVEAVHAWRNALTGAPLN |
| --- | --- |
| **Cter Sequence (with FokI)** | SIVAQLSRPDPALAALTNDHLVALACLGGRPALDAVKKGLGDPISRSQLVKSELEEKKSELRHKLKYVPHEYIELIEIARNSTQDRILEMKVMEFFMKVYGYRGKHLGGSRKPDGAIYTVGSPIDYGVIVDTKAYSGGYNLPIGQADEMQRYVEENQTRNKHINPNEWWKVYPSSVTEFKFLFVSGHFKGNYKAQLTRLNHITNCNGAVLSVEELLIGGEMIKAGTLTLEEVRRKFNNGEINFAAA |

TALEN array RVD composition and target sequences used for the Tail to Head (TtT) architectures.

| **Locus** | **RVD Array compositions** | **Target Sequence** |
| --- | --- | --- |
| **APC** | NN-NN-HD-NG-NN-HD-NI-NN-HD-NG-NG-HD-NI-NG-NI-NG#  NG-HD-NI-NN-NG-NN-HD-HD-NG-HD-NI-NI-HD-NG-NG-NG# | GGCTGCAGCTTCATAT  TCAGTGCCTCAACTTG |
| **MLH1** | NG-HD-NN-NG-NN-NN-HD-NI-NN-NN-NN-NN-NG-NG-NI-NG#  NN-HD-NN-NN-NG-NG-HD-NI-HD-HD-NI-HD-NG-NN-NG-NG# | TCGTGGCAGGGGTTAT  GCGGTTCACCACTGTC |
| **NR3C3** | NN-NN-NG-NN-NG-NN-NI-NN-NG-NI-HD-HD-NG-HD-NG-NG#  NN-NG-NI-NG-NG-HD-NI-NG-NN-NG-HD-NI-NG-NI-NN-NG# | GGTGTGAGTACCTCTG  GTATTCATGTCATAGT |
| **BBC3** | NG-NN-NN-NN-NG-NN-NI-NN-NI-HD-HD-HD-NI-NN-NG-NG#  NN-NN-NI-NG-NG-HD-HD-NG-NN-NG-HD-NG-HD-HD-NG-NG# | TGGGTGAGACCCAGTA  GGATTCCTGTCTCCTC |
| **LIG4** | NN-HD-NG-NG-NN-HD-NG-NI-NI-NN-HD-NG-NG-NG-NI-NG#  NG-NG-HD-HD-NI-NG-HD-NG-HD-NG-NI-NN-NN-NG-NI-NG# | GCTTGCTAAGCTTTAT  TTCCATCTCTAGGTAA |
| **M2K** | NG-NN-HD-NG-NN-HD-NI-NG-NG-NN-HD-NI-NN-HD-HD-NG#  NN-NG-NG-NN-NN-NN-NI-NN-NG-NN-NI-NI-NN-NI-NN-NG# | TGCTGCATTGCAGCCG  GTTGGGAGTGAAGAGC |
| **ERBB2** | NN-HD-NG-NG-NN-NI-NN-NN-NI-NI-NN-NG-NI-NG-NI-NG#  NN-NN-NI-NN-NN-NN-NN-NI-NI-NG-HD-NG-HD-NI-NN-NG# | GCTTGAGGAAGTATAA  GGAGGGGAATCTCAGC |
| **PPARD** | NN-HD-NI-NG-NN-NG-NN-NI-NN-NN-NN-NN-NG-NN-HD-NG#  NN-NN-HD-HD-NI-NN-HD-HD-NI-HD-HD-NN-HD-NG-HD-NG# | GCATGTGAGGGGTGCA  GGCCAGCCACCGCTCC |
| **NR3C2** | NN-NN-NG-NI-NG-NG-HD-NI-HD-NG-NI-NI-NG-HD-NG-NG#  NN-HD-NI-NG-NN-NG-NI-NI-NI-NN-HD-NG-NN-HD-NI-NG# | GGTATTCACTAATCTG  GCATGTAAAGCTGCAG |
| **CD52** | NN-NN-NN-NI-NN-NN-NN-NN-NG-NG-NN-NI-NG-NN-HD-NG#  NN-HD-HD-NG-NN-NG-HD-NI-NI-HD-NG-NG-HD-NG-NI-NG# | GGGAGGGGTTGATGCC  GCCTGTCAACTTCTAC |

**Supplementary table 7**

Induced mutagenesis frequencies found with the classical Tail to Tail (TtT) architecture.

| **Locus** | | | **Total Events** | **Total Events** | **Insertion** | **Deletion** | **wt** | **Reads** |
| --- | --- | --- | --- | --- | --- | --- | --- | --- |
| **Cell line** | **Name** | **Target** | **[%]** | **[nb]** | **[nb]** | **[nb]** | **[nb]** | **[nb]** |
| 293H | APC | TGGCTGCAGCTTCATATGATCAGTTGTTAAAGCAAGTTGAGGCACTGAA | **3.67** | 50 | 17 | 33 | 1314 | 1390 |
| 293H | MLH1 | TTCGTGGCAGGGGTTATTCGGCGGCTGGACGAGACAGTGGTGAACCGCA | **33.76** | 2471 | 320 | 2198 | 4849 | 7367 |
| 293H | NR3C3 | TGGTGTGAGTACCTCTGGAGGACAGATGTACCACTATGACATGAATACA | **0.49** | 22 | 14 | 8 | 4443 | 4567 |
| 293H | BBC3 | TTGGGTGAGACCCAGTAAGGATGGAAAGTGTAGAGGAGACAGGAATCCA | **20.31** | 1045 | 196 | 868 | 4100 | 5168 |
| 293H | LIG4 | TGCTTGCTAAGCTTTATATTGAGTTGCTTAATTTACCTAGAGATGGAAA | **19.92** | 1422 | 167 | 1277 | 5718 | 7148 |
| 293H | M2K | TTGCTGCATTGCAGCCGCCGCGGCGCCGCTCGGCTCTTCACTCCCAACA | **31.52** | 185 | 48 | 139 | 402 | 14838 |
| 293H | ERBB2 | TGCTTGAGGAAGTATAAGAATGAAGTTGTGAAGCTGAGATTCCCCTCCA | **34.77** | 1340 | 256 | 1112 | 2514 | 3955 |
| 293H | PPARD | TGCATGTGAGGGGTGCAAGGTACGGACTGGGGGGAGCGGTGGCTGGCCA | **1.39** | 58 | 24 | 35 | 4115 | 5237 |
| 293H | NR3C2 | TGGTATTCACTAATCTGGGAAGGGAAGGGCTACTGCAGCTTTACATGCA | **3.83** | 375 | 104 | 278 | 9427 | 9810 |
| 293H | CD52 | TGGGAGGGGTTGATGCCAGACATCACCAGGTTGTAGAAGTTGACAGGCA | **3.46** | 103 | 57 | 47 | 2877 | 3334 |

**Supplementary table 8**

Sequences of oligos used for amplification of endogenous sites.

|  | **Oligo For (5’🡪3’)** | **Oligo Rev (5’🡪3’)** |
| --- | --- | --- |
| **RAG1 caaccaaccccctggaagactg gtgggtgctgaatttcatctgg**  **DMDT2 cctgatatttctcctattaatattg GGAGTGTGGTACTTCATCATGTCAGA**  **FUT8T3 TTTCTCTGGAAGAATCCCAAGG AAATCTGTCCCTAGAAGTGTGC** | caaccaaccccctggaagactg | gtgggtgctgaatttcatctgg |
| **DMD** | cctgatatttctcctattaatattg | GGAGTGTGGTACTTCATCATGTCAGA |
| **FUT8** | TTTCTCTGGAAGAATCCCAAGG | AAATCTGTCCCTAGAAGTGTGC |
| **APC** | ccactgtttcatcctcttagatgc | gataccttcatattagatgcctcag |
| **MLH1** | gtccaatcaatagctgccgctgaag | tgggcatgcgctgtacatgcctc |
| **NR3C3** | cagagtccccagagaagtcaag | tcatcgaactctgcacccctgg |
| **BBC3** | ctgtctcatagctttccattccg | gagtgtgtacttggaggcagtc |
| **LIG4** | ggtcgtttacttgctgtatgg | gatggctgcctcacaaacttcac |
| **M2K** | caagacagctgtctgcttcacagg | gttccttaccctgcatgctgctgac |
| **ERBB2** | gtgaagctgggagttgccactcc | ccagctccatggtgctcactgcg |
| **PPARD** | catcgtgtgtccgcagacctctc | tcctgcagtgcccagagctgagg |
| **NR3C2** | gaccatcgctgcctgtatgaatg | gctgtagtagccctttctgtgtgc |
| **CD52** | cgtggccaatgccataatccacc | gtagggatgtccagtaaccacaag |
